# Factors Associated With Lameness in Tie Stall Housed Dairy Cows in South Germany

**DOI:** 10.3389/fvets.2020.601640

**Published:** 2020-12-16

**Authors:** Andreas W. Oehm, Katharina Charlotte Jensen, Annegret Tautenhahn, Kerstin-Elisabeth Mueller, Melanie Feist, Roswitha Merle

**Affiliations:** ^1^Clinic for Ruminants With Ambulatory and Herd Health Services, Ludwig-Maximilians Universität Munich, Munich, Germany; ^2^Clinic for Cattle, Foundation, University of Veterinary Medicine, Hanover, Germany; ^3^Clinic for Ruminants and Swine, Faculty of Veterinary Medicine, Freie Universität Berlin, Berlin, Germany; ^4^Institute for Veterinary Epidemiology and Biostatistics, Freie Universität Berlin, Berlin, Germany

**Keywords:** locomotion, cattle, risk factor analysis, housing conditions, tie stall, lameness

## Abstract

Lameness remains a major concern for animal welfare and productivity in modern dairy production. Even though a trend toward loose housing systems exists and the public expects livestock to be kept under conditions where freedom of movement and the expression of natural behavior are ensured, restrictive housing systems continue to be the predominant type of housing in some regions. Factors associated with lameness were evaluated by application of multiple logistic regression modeling on data of 1,006 dairy cows from 56 tie stall farms in Bavaria, South Germany. In this population, approximately every fourth cow was lame (24.44% of scored animals). The mean farm level prevalence of lameness was 23.28%. In total, 22 factors were analyzed regarding their association with lameness. A low Body Condition Score (BCS) (OR 1.54 [95%-CI 1.05–2.25]) as well as increasing parity (OR 1.41 [95%-CI 1.29–1.54]) entailed greater odds of lameness. Moreover, higher milk yield (OR 0.98 [95%-CI 0.96–1.00]) and organic farming (OR 0.48 [95%-0.25–0.92]) appeared to be protectively associated with lameness. Cows with hock injuries (OR 2.57 [95%-CI 1.41–4.67]) or with swellings of the ribs (OR 2.55 [95%-CI 1.53–4.23]) had higher odds of lameness. A similar association was observed for the contamination of the lower legs with distinct plaques of manure (OR 1.88 [95%-CI 1.14–3.10]). As a central aspect of tie stall housing, the length of the stalls was associated with lameness; with stalls of medium [(>158–171 cm) (OR 2.15 [95%-CI 1.29–3.58]) and short (≤158 cm) length (OR 4.07 [95%-CI 2.35–7.05]) increasing the odds compared with long stalls (>171 cm). These results can help both gaining knowledge on relevant factors associated with lameness as well as approaching the problem of dairy cow lameness in tie stall operations.

## Introduction

Lameness, defined as impaired locomotion regardless of the underlying cause (1–3), is the most important matter for economic and animal welfare concerns in modern dairy production (4–8). It has considerable adverse effects on longevity, milk yield, reproductive performance, and general well-being (9–12). Although muscle damage and nerve paralysis contribute to lameness, by far the most cases originate from claw disorders (13). While the source of pain in the initial phase of a claw disorder is the lesion itself, hyperalgesia is present in chronic cases, which does not need to be related to the severity of the lesion (14–16). Painful disorders impair the natural behavior of affected animals (16–19). Lameness is multifactorial by origin with housing conditions, on-farm management practices, and the individual animal having the greatest impact (20, 21).

Even though modern dairy husbandry has been experiencing a shift toward loose housing systems, keeping dairy cows in tie stall facilities is still a common husbandry method worldwide (8, 22, 23). This practice has yet been criticized due to increasing concerns of consumers about the well-being and quality of life of livestock (24, 25). Even though tie stall housing often incorporates pasture access, animals are mostly restrained in their individual stalls throughout their productive life, they are unable to move freely or express natural behavioral patterns. Concerning lameness however, lower prevalence has been reported for tie stall facilities compared with free stall barns (26).

The aim of the present study was to assess the prevalence of lameness in tie stall housed dairy cows in South Germany and to evaluate the association of lameness with potential risk factors.

## Materials and Methods

### Farm Recruitment

This study was conducted as part of a large cross-sectional study on health, biosecurity, and housing environment on dairy farms in Germany. The project was initiated and funded by the German Federal Ministry of Food and Agriculture (BMEL) through the Federal Office for Agriculture and Food (BLE) grant number 2814HS008. A total amount of 265 dairy farms in the German federal state of Bavaria were visited. Farms were randomly selected stratified by administrative district and farm size within their federal states. Sampling was based on the national animal information data base (HIT) and on the farm data from the Milchprüfring Bayern e.V. Selected farms received a letter including information on the study and an invitation to participate. Interested farmers contacted the study team voluntarily and gave their written consent to participate in the study. Farms were visited once between December 2016 and March 2019. In the present study, farms housing their cows in tie stall facilities were included.

### On-Farm Data Collection

Inter-observer reliability between all of the seven researchers collecting the data was assessed three times during the study period. Each of these assessments took place in the form of a 2 day practical course. During the first assessment, 44 cows were scored, 59 cows were scored during the second assessment, and 73 cows were scored at the third assessment date. Furthermore, video as well as photo material was evaluated in group discussions conducted after each of the meetings. Based on these assessments, a weak/moderate, substantial, and fair agreement was present between the observers (overall weak to moderate, kappa values of 0.57, 0.63, and 0.44, respectively) (27, 28).

On each farm, all cows were assessed. The individual ear tag number of the animals (last five digits) was documented. All lactating and dry cows that were tied at the day of the farm visit individually underwent scoring for lameness, body condition, rib swellings, cleanliness of the lower legs and udder, and the presence of observable abnormalities of the hock, neck, back, and tail.

Lameness was assessed using the Stall Lameness Score (SLS) introduced by Leach et al. (29). Four criteria were observed during a 90 s observation period: weight shifting between feet, sparing a foot while standing, unequal weight bearing when stepping from side to side, and standing on the edge of the kerb (29). A cow displaying two out of the four criteria patterns was classified as lame. Body condition was scored according to the Body Condition Score (BCS) established by Edmonson et al. (30), later modified by Metzner et al. (31). As body condition changes during lactation, breed-specific categories exist in regard to days in milk. Therefore, cows were assigned to one of the three body condition categories “under,” “opt,” and “over” in relation to breed and stage of lactation (32–34), which can be seen in Table 1.

**Table 1:** BCS categories in accordance with stage of lactation and breed (32–34).

**Days in milk**	**Breed**								
	**Holstein**			**Brown swiss**			**Simmental/other**		
	**Under**	**Optimal**	**Over**	**Under**	**Optimal**	**Over**	**Under**	**Optimal**	**Over**
0–29	≤ 2.75	3.0–3.75	> 3.75	≤ 2.75	3.0–3.75	> 3.75	≤ 3.25	3.5–4.25	> 4.25
30–99	≤ 2.5	2.75–3.25	> 3.25	≤ 2.5	2.75–3.25	> 3.25	≤ 3.0	3.25–4.0	> 4.0
100–199	≤ 2.5	2.75–3.25	> 3.25	≤ 2.5	2.75–3.25	> 3.25	≤ 3.0	3.25–4.0	> 4.0
200–299	≤ 2.75	3.0–3.75	> 3.75	≤ 2.75	3.0–3.75	> 3.75	≤ 3.25	3.5–4.25	> 4.25
> 300	< 3.25	3.25–3.75	> 3.75	< 3.25	3.25–3.75	> 3.75	< 3.75	3.75–4.25	> 4.25

The presence of rib swellings was visually assessed in the lateral thoracic region between the 7 and the 9th rib at the transition from the bony part to the cartilaginous part of the rib (35).

A modified scoring approach was implemented to record skin changes of the hock (36, 37). Accordingly, hocks were assessed from a caudolateral perspective as follows: 1 = no skin change, 2 = hairless patch, 3 = swelling (no wound), 4 = wound (no swelling), 5 = wound and swelling, 6 = no assessment possible due to solid plaque of manure. The most severe of the present abnormalities on both sides was recorded. Skin changes of the neck were documented if present in the region between the first cervical and the first thoracic vertebra. A modified score according to Kielland et al. (38) was implemented: 1 = no observable skin change, 2 = hairless patch, 3 = wound or swelling. To assess the back, the region between the first cervical and the first caudal vertebra in an area of 10 cm on both sides of the median line of the back was examined. As for the tail, only visible abnormalities were documented: 1 = no abnormalities, 2 = swelling or deviation of the tail, 3 = amputated tail. Cleanliness of the udder and the lower legs was appraised according to Cook and Reinemann (39): 1 = little or no manure, 2 = minor splashing, 3 = distinct plaques of manure, 4 = solid plaque of manure.

The type of tying system, type of stall base, use of bedding material, and gutter design were assessed by visual inspection. An a priori determined number of stanchions per farm was measured for length and width: up to 30 stanchions with cows: 10 stanchions were measured; 30–49 stanchions: 15 stanchions were measured; 50–99 stanchions: 17 stanchions were measured. This number had been calculated prior to farm visits in accordance with farm size (i.e., the number of stalls present on farm in this context). For example, if 30 stanchions were present on farm and 10 had to be measured according to the pre-defined plan, every 3rd stall was assessed. The median value per farm was calculated and used for further statistical analysis.

Farmers were interviewed during the farm visit in order to collect information on the operational type of the farm (main source of income, organic farming) and if cows were provided with access to pasture or an outdoor exercise area at any given time during the year. Data on milk yield, parity, age, breed, and days in milk were retrieved from the national animal information data base HIT and from the national milk recording system (DHI). Farm records for milk yield were available for each cow up to 12 months prior to the farm visit. Test day milk yield is assessed once a month. In the present study, the most recent test day milk yield was used.

### Data Handling and Statistical Analysis

All data were collected using questionnaires and data entry forms. After the farm visit, data were manually entered into a central SQL-data base. From there Microsoft Excel (40) datasheets were extracted and imported into R.

Statistical analyses were conducted with the statistical software R version 1.2.1335 (41). We used the following five packages: tidyverse (42), ggstatsplot (43), sjPlot (44), effects (45), and caret (46).

Descriptive statistics were carried out to investigate the distribution of predictors with the Stall Lameness Score. Abnormalities of the back and the tail were dichotomized. As for hocks, all cows that scored 6 were excluded from further analyses. Moreover, observable skin changes of the hocks were further categorized to 1 (no observable skin changes present), 2 (hairless patches), and 3 (swelling and/or wound). The continuous variables *stall length* and *stall width* were transformed into categorical variables depending on their distribution and the values of their quartiles. Three categories were created: short (≤ 158 cm), medium (>158–171 cm), and long (>171 cm). Farm size was grouped into three categories: small (<24 cows), medium (24–30 cows), and large (>30 cows). Subsequently, univariable analyses were performed on cow level for each variable in regard to the targeted variable *lame* (1/0) using logistic regression. A *p* ≤ 0.05 was regarded as statistically significant.

Multiple mixed logistic regression models were built on cow level in a manual stepwise forward selection process adding one predictor at a time to the model; *farm* was included as random factor. *Year* and *farm size* (categorized) were included as fixed effects. After every newly included variable, the model was assessed using the Akaike's Information (AIC) and Conditional *R*^2^. The lower the AIC the better the quality of the model (47). If a significant improvement of the AIC was perceived, a variable was kept within the model. Furthermore, after each step, the R function car::vif() was implemented for variable inflation in order to detect potential (multi-)collinearity among predictors.

## Results

A total number of 1,170 dairy cows on 56 farms in the south German state of Bavaria were included in the data set of this study based on the housing system of their cows. If cows were housed in tie stalls at farm visit, these farms were included in the present analysis which led to the inclusion of 56 farms out of the initial 265 farms. The mean farm size was 25.60 cows (range 4–61 animals). Of the 56 farms, 47 were run conventionally whereas 9 farms were managed according to principles of organic farming. The predominant breed was German Simmental (84.53%), followed by Brown Swiss (10.77%), German Holstein (2.65%) and others (2.05%), i.e., crossbreds of the aforementioned. On 33 farms, dairy farming was the main source of income, whereas dairy farming provided subsidiary income on 23 farms. Among the 1,170 cows, 286 were classified as *lame* which equals a lameness prevalence of 24.44%. On farm level, the mean lameness prevalence was 23.28% (5.26–51.58%). Descriptive statistics of all categorical variables within the data set are presented in Table 2. Descriptive statistics of numerical variables within the data set can be seen in Table 3.

**Table 2:** Distribution of categorical variables within the data set.

**Predictor**	**Categories**	**n _**cows**_ (%)**
Breed	German Simmental other	989 (84.53) 181 (15.47)
Udder hygiene	1 (little or no manure) 2 (minor splashing) 3 (distinct plaques of manure) 4 (solid plaque of manure)	344 (29.40) 405 (34.62) 246 (21.03) 175 (14.96)
Cleanliness of lower legs	1 (little or no manure) 2 (minor splashing) 3 (distinct plaques of manure) 4 (solid plaque of manure)	357 (30.51) 519 (44.36) 199 (17.01) 95 (8.12)
Hock	1 (no observable skin change) 2 (hairless patch) 3 (swelling and/ or wound)	160 (15.90) 604 (60.04) 242 (24.06)
Swelling of the ribs	No Yes	1,072 (91.62) 98 (8.38)
Neck	1 (no observable skin change) 2 (hairless patch) 3 (swelling and/ or wound)	603 (51.54) 473 (40.43) 94 (8.03)
Back	0 (no observable skin change) 1 (skin change present)	1,133 (96.84) 37 (3.16)
Tail	0 (no observable skin change) 1 (skin change present)	1,103 (94.43) 65 (5.57)
Income from dairy farming	Main income Subsidiary income	794 (69.22) 353 (30.78)
BCS	Underconditioned Optimally conditioned Overconditioned	262 (22.39) 824 (70.43) 84 (7.12)
Type of tying system	Grabner tie^a^ Vertical neck frame Collar and chain Other	713 (62.65) 150 (13.18) 198 (17.40) 77 (6.77)
Stall base	Concrete Rubber	181 (16.20) 936 (83.80)
Use of bedding	No Yes	1,137 (97.26) 32 (2.74)
Gutter design	Concrete Grate	205 (18.22) 920 (81.78)
Farming type	Conventional farming Organic farming	1,006 (86.00) 164 (14.00)
Access to pasture	No Yes	718 (61.37) 452 (38.63)
Exercise area present	No Yes	1,035 (88.46) 135 (11.54)
Length of stalls (categorized)^b^	1 (short) 2 (medium) 3 (long)	291 (24.87) 539 (46.07) 340 (29.6)
Width of stalls (categorized)^c^	1 (narrow) 2 (medium) 3 (broad)	318 (28.14) 519 (45.93) 293 (25.93)
Farm size (categorized)^d^	1 (small) 2 (medium) 3 (large)	583 (49.83) 282 (24.10) 305 (26.07)
Observer	1 2 3 4 5	132 (11.28) 331 (28.29) 85 (7.26) 113 (9.66) 274 (23.42)
	6	126 (10.77)
	7	109 (9.32)

*n_cows_: absolute number of cows*.

a *chain/belt fixed vertically with attached sliding frame around the cow's neck*.

b *length of stalls was categorized according to the distribution of the measured values and the medians calculated from these (≤ 158 cm: 1; > 158–171 cm: 2; > 171 cm: 3)*.

c *width of stalls was categorized according to the distribution of the measured values and the medians calculated from these (≤ 98.5 cm: 1; > 98.5–103 cm: 2; > 103 cm: 3)*.

d *farm size was categorized (small < 24 cows; medium 24–30 cows; large > 30 cows)*.

**Table 3:** Distribution of continuous variables within the data set.

**Predictor**	**Mean**	**Range**	**1st quartile**	**Median**	**3rd quartile**	***n***
Parity	2.71	1–11	1	2	4	1,170
Days in milk	200.35	0–1,060	92	192	284	1,170
Milk yield (in kg)^*^	22.91	4.80–51.80	17.12	22.30	28.10	1,170
Farm size	25.60	4.00–61.00	19.00	24.00	30.00	1,170

* *Variable on cow level; values obtained from the most recent sampling record*.

Table 4 summarizes the results of the univariable analyses. All predictors were analyzed in relation to the outcome *lame*.

**Table 4:** Results of the univariable analyses of all factors with the target variable *lame*.

**Predictor**	**Parameter estimate**	**Standard error**	**Odds ratio**	**Confidence interval (95%)**	***P*-value**
**Breed**					
Other German Simmental	Reference 0.08	–0.19	–1.08	–0.75–1.59	– 0.673
**Parity**					
Increasing parity	0.26	0.04	1.30	1.21–1.39	< 0.001
Days in Milk	0.00045	0.00049	1.00	1.00–1.00	0.353
Milk yield	−0.00062	0.0087	1.00	0.98–1.02	0.943
**Udder hygiene**					
Little/no manure Minor splashing Distinct plaques of manure Solid plaque of manure	Reference 0.12 0.08 0.45	–0.170.190.21	− 1.13 1.09 1.57	–0.80–1.590.74–1.601.04–2.37	– 0.497 0.671 0.030
**Cleanliness of lower leg**					
Little/no manure Minor splashing Distinct plaques of manure Solid plaque of manure	Reference 0.20 0.52 0.54	–0.170.200.26	– 1.22 1.68 1.71	–0.88–1.701.13–2.501.02–2.82	– 0.231 0.011 0.038
**Hocks**					
No observable skin change Hairless patch Swelling and/or wound	Reference 0.50 1.31	–0.240.26	– 1.65 3.73	–1.04–2.702.28–6.28	– 0.039 < 0.001
**Swelling of the rib**					
No Yes	Reference 1.12	–0.22	– 3.07	–2.01–4.68	– < 0.001
**Neck**					
No observable skin change Hairless patch swelling or wound	Reference 0.51 0.18	–0.140.26	– 1.66 0.70	–1.25–2.191.97	– < 0.001 0.505
**Back**					
No observable skin chance Skin change present	Reference 1.00	–0.34	– 2.73	–1.39–5.29	– 0.003
**Tail**					
No observable abnormality Deviation and/or swelling, amputated tail	Reference −0.27	–0.32	– 0.76	–0.39–1.38	– 0.397
**Income from dairy farming**					
Main income Subsidiary income	Reference −0.05	–0.15	– 0.95	–0.71–1.28	– 0.756
**BCS**					
Underconditioned Optimally conditioned Overconditioned	0.58 Reference 0.32	0.16–0.26	1.79 – 1.38	1.31–2.42–0.82–2.26	< 0.001 – 0.215
**Type of tying system**					
Grabner tie^a^ Vertical neck frame Collar and chain Other	Reference −0.47 −0.59 −1.24	–0.220.200.38	– 0.62 0.55 0.29	–0.40–0.950.37–0.820.13–0.58	– 0.032 0.004 0.001
**Stall base**					
Concrete Rubber	Reference - 0.05	–0.19	– 0.95	–0.66–1.39	– 0.791
**Use of bedding**					
No bedding Bedding present	Reference 0.03	–0.41	– 1.03	–0.43–2.22	– 0.943
**Gutter design**					
Concrete or gutter without grate Gutter with grate	Reference 0.52	–0.20	– 1.69	–1.15–2.52	– 0.008
**Farming type**					
Conventional farming Organic farming	Reference −0.77	–0.24	– 0.46	–0.28–0.72	– 0.001
**Access to pasture**					
No Yes	Reference −0.58	–0.15	– 0.56	–0.42–0.74	– < 0.001
**Exercise area present**					
No Yes	Reference −0.56	–0.24	– 0.57	–0.34–0.90	– 0.021
**Length of stalls (categorized)^b^**					
1 (short) 2 (medium) 3 (long)	1.24 0.77 Reference	0.210.20–	3.45 2.16 –	2.31–5.241.47–3.24–	< 0.001 < 0.001 –
**Width of stalls (categorized)^c^**					
1 (narrow) 2 (medium) 3 (broad)	Reference 0.005 −0.49	–0.160.20	– 1.01 0.61	–0.73–1.380.41–0.90	– 0.975 0.013
**Farm size (categorized)^d^**					
1 (small) 2 (medium) 3 (large)	Reference 0.15 0.71	–0.180.16	– 1.16 2.04	–0.82–1.631.48–2.78	– 0.407 < 0.001
**Year**					
2016 2017 2018 2019	Reference −0.59 −1.01 −1.21	–0.280.290.35	– 0.55 0.37 0.30	–0.32–0.960.21–0.640.15–0.59	– 0.034 < 0.001 0.001
**Observer**					
1 2 3 4 5 6 7	Reference 0.11 −0.53 −0.06 −0.22 −0.35 0.55	–0.240.350.300.250.300.28	– 1.12 0.59 0.94 0.81 0.71 1.74	–0.71–1.800.29–1.170.52–1.690.50–1.320.39–1.271.00–3.04	– 0.630 0.139 0.841 0.386 0.2501 0.053

a *Chain/belt fixed vertically with attached sliding frame around the cow's neck*.

b *Length of stalls was categorized according to the distribution of the measured values and the medians calculated from these (≤ 158 cm: 1; > 158–171 cm: 2; > 171 cm: 3)*.

c *Width of stalls was categorized according to the distribution of the measured values and the medians calculated from these (≤ 98.5 cm: 1; > 98.5–103 cm: 2; > 103 cm: 3)*.

d *Farm size was categorized (small < 24 cows; medium 24–30 cows; large > 30 cows)*.

The multiple logistic regression approach required a complete cases data set. Accordingly, missing observations were removed which resulted in a total number of 1,006 cows on 56 farms. The final model maintained 8 out of the 22 predictors as well as the fixed effects *year* and *farm size* (categorized). Table 5 displays an overview of the results from the final multiple mixed logistic regression model. Low BCS was associated with greater odds of lameness. Compared with optimally conditioned cows, underconditioned animals experience higher odds of being lame (OR 1.59 [CI 1.10–2.30], *p* = 0.014). Higher odds of lameness were observed in animals of parities 3 or higher compared with animals in their first lactation (OR 2.71 [CI 1.83–4.01], *p* < 0.001). Furthermore, increasing milk yield was associated with lameness (OR 0.98 [CI 0.96–1.00], *p* = 0.05). With increasing levels of contamination of the lower legs, cows experienced higher odds of lameness. This was noticeable for the presence of distinct plaques of manure (OR 1.61 [CI 1.00–2.61], *p* = 0.05), but not for solid plaques of manure (OR 1.30 [CI 0.66–2.57], *p* = 0.443). Swellings and/or open wounds in the hock region were associated with lameness (OR 2.56 [CI 1.43–4.61], *p* = 0.002) as well as the presence of rib swellings (OR 2.81 [1.70–4.64], *p* < 0.001). Compared with long stalls, cows kept in medium (OR 1.76 [CI 1.07–2.87], *p* = 0.025) or short (OR 3.17 [CI 1.93–5.19], *p* < 0.001) stalls appeared to have greater odds of lameness. Cows living on farms with more than 30 cows have higher odds for lameness compared with cows on small farms (<24 cows) (OR 1.72 [CI 1.15–2.58], *p* = 0.008). As animals on different farms are not subjected to the same housing and management conditions, a farm-specific random effect was introduced in the modeling procedure in order to account for the presence of random variability in the data due to actual differences in on-farm housing- and management practices. The random effect considered that effects may differ as a consequence of differences across farms and incorporates farm-to-farm-variability within the analysis. In the current study, the percentage of heterogeneity, i.e., the value of τ_00__farm_ as the variance between farms, in the final model was 0.20. Hence, 20% of the variance were explained by the variance between farms, e.g., as a consequence of different settings, varying housing conditions, management elements or of a different mindset.

**Table 5:** Final multiple logistic regression model for factors associated with lameness.

			***Lame***		
**Predictor**	**Category**	**Parameter estimate**	**Odds ratio**	**Confidence interval (95%)**	***P*-value**
Intercept		−1.82	0.16^***^	0.06–0.43	< 0.001
BCS	Optimal	Reference	–	–	–
	Overconditioned	−0.14	0.87	0.48–1.59	0.656
	Underconditioned	0.46	1.59^*^	1.10–2.30	0.014
Parity	1	Reference	–	–	–
	2	0.0021	1.00	0.62–1.61	0.993
	≥ 3	1.00	2.71^***^	1.83–4.01	< 0.001
Milk yield	Continuous^a^	−0.02	0.98^*^	0.96–1.00	0.05
Leg cleanliness	Little/no manure	Reference	–	–	–
	Minor splashing	0.03	1.03	0.71–1.50	0868
	Distinct plaques of manure	0.47	1.61^*^	1.00–2.61	0.05
	Solid plaque of manure	0.94	1.30	0.66–2.57	0.443
Hocks	No observable skin change	Reference	–	–	–
	Hairless patch	0.26	1.30	0.76–2.20	0.338
	Swelling and/or wound	0.94	2.56^**^	1.43–4.61	0.002
Rib swelling	No	Reference	–	–	–
	yes	1.03	2.81^***^	1.70–4.64	< 0.001
Farming type	Conventional farming	Reference	–	–	–
	Organic farming	−0.46	0.63	0.35–1.14	0.125
Length of stalls	Long	Reference	–	–	–
	Medium	0.56	1.76^*^	1.07–2.87	0.025
	Short	1.15	3.17^***^	1.93–5.19	< 0.001
Farm size	Small Medium Large	Reference0.290.55	– 1.34 1.72	–0.87–2.081.15–2.58	– 0.189 0.008
Year	2016 2017 2018 2019	Reference−0.89−0.85−0.84	– 0.41 0.43 0.43	–0.20–0.820.22–0.840.19–0.96	– 0.012 0.014 0.040

*Out of the initial 22 predictors, 10 factors associated with housing conditions and the individual animal were maintained within the final model. The model incorporated data from 1,006 dairy cows on 56 farms*.

a *1 unit increase*.

* *p < 0.05*,

** p < 0.01, and

*** *p < 0.001*.

## Discussion

As public interest in the welfare and physical integrity of agricultural livestock in modern production systems grows, husbandry conditions are likely to come under close scrutiny which necessitates a critical evaluation in order to both meet animal welfare standards and economic viability (48). This growing public focus on farm animal welfare requires further investigation in current practices and to broaden our knowledge concerning housing conditions of livestock. This is particularly important with regard to lameness prevalence which is often addressed in the context of welfare assessment (49, 50). Against this background, the aim of this study was to determine the prevalence of lameness in tie stall housed dairy cows in Bavaria and to evaluate factors associated with the condition. By including a large number of animals and farms, we are convinced to have attained a high level of standardization even though some limitations exist. The mean farm level prevalence of lameness was 23.28 and 24.44% on animal level which is similar to other studies. In a Canadian study, Bouffard et al. (51) also implemented the SLS to determine lameness prevalence and found 25% of the cows assessed to be lame. In general, lameness prevalences are higher in free stall facilities than in tie stall operations and other housing types (6, 52, 53). Regarding the lameness prevalence determined in the current study, it is important to acknowledge, that Leach et al. (29) only observed a moderate sensitivity (0.54–0.77) of the SLS in direct comparison with locomotion scoring according to Sprecher et al. (54). This means that lameness might be underestimated when detected by SLS. The prevalence of lameness was underestimated on average by 27% (11–37%) in the study by Leach et al. (29). Moreover, as farmers had to get in contact with the study team on their own initiative, one might infer that mainly proactive farmers or well-conditioned operations have been enrolled and visited. This circumstance raises the hypothesis that the true lameness prevalence could be even higher in the dairy cow population housed in tie stall facilities. On the other hand, it appears plausible to assume that voluntary participation may have motivated specifically those farms with a lameness problem to participate. In this case, the true lameness prevalence in the current study would be overestimated.

Cows with a BCS lower than recommended (32–34) had higher odds of lameness compared with cows with a higher BCS according to breed and stage of lactation. This association is in accordance with others (20, 55, 56). As loss in body condition is not exclusively related to subcutaneous body fat but also affects the digital cushion, its shock absorbing properties during weight-bearing are impaired exposing the sensitive structures of the claw, i.e., the distal phalanx and the corium to undissipated mechanical forces that subsequently result in the formation of traumatic claw lesions (56–58). On the other hand, lameness itself often entails a loss of body condition as animals show alterations in their feeding behavior (59–61). Regarding the BCS limits in the present study, Holstein cows where considered as optimally conditioned with a BCS of up to 3.75 at the start of lactation as well as in the later stages of lactation and during the dry period. These cut offs where selected in accordance with the above cited literature. It is yet important to be aware that Drackley (62) recommended that BCS may not exceed 3.0 in North American Holstein cows at the beginning of lactation. As Holstein cows represented only a minor part of the study population in the present study and since difference might be present between Holstein cows of the North American type and the European or German type, respectively, we decided to implement the values presented in European publications that also provided cut off values for other breeds of the study population. As outlined previously, the results regarding the association between BCS and lameness are well in accordance with previous work. Using the stricter cut off values for Holstein cows suggested by Drackley (62), the result may have become even more distinct.

Higher parity increased a cow's odds for lameness in the current study and in previous work (63, 64). Prolonged exposition to potentially harmful elements of housing and management environments may increase the odds for cows higher in parity to suffer from recurrent episodes of claw disorders, finally resulting in chronic lameness (63–66). Older animals may also be less able to cope with deficient housing conditions. Furthermore, the tensile strength of the suspensory apparatus progressively wears out with increasing parity which causes the third phalanx to remain in a state of sinking (65, 67, 68). In combination with delivery associated remodeling processes of both the suspensory apparatus of the claw and the digital cushion, the deeper, more sensible structures of the claw may experience impaired shock-absorbing capacity and hence a massive increase of pressure (57, 58, 65, 69, 70). This subsequently fosters the development of traumatic claw lesions and lameness. On the other hand, dairy cows in their first lactation may encounter the most pronounced problems with housing associated changes when they are transferred from a heifer group to the group of lactating animals. The transition from free housing as heifer to tied housing as a lactating cow may create challenges for these animals and they may hence be removed from the herd prematurely which is supported by the fact that dairy cows in Germany survive to an average age of 5.4 years (71–73). This in sharp contrast to the aspiration of keeping dairy cows for a long productive life and highlights the fact that the current housing systems ought to be re-considered in order to be adequate to keep the animals sound and physically intact on the long run. It furthermore emphasizes that with increasing parity cows need to be provided with special care.

The association between high milk yield and the occurrence of disease, e.g., lameness, cows has been subject to ongoing discussions (74–76) with high yielding animals being particularly at risk for metabolic disorders, reproductive deficiencies, and lameness (77, 78). In tie stalls in southern Germany, cows are mostly fed with single components instead of mixed rations provided in free stall barns. Therefore, it is difficult to meet the nutritional requirements of high-yielding cows. Counterintuitively, high milk yield appeared to reduce the odds of lameness in the current study (OR 0.98 [0.95–1.00]) which is also confirmed by investigations made by Wangler et al. (73). This may be explained by the fact that cows with a high milk yield may be exposed to improved management and housing procedures which keep animals in a healthy condition (and consequently being less lame) and enable them to meet their productive potential. Another reason might be that lame cows cannot reach their full potential due to changed feeding behavior and inflammation processes (9, 79). It is importance to note that according to Green et al. (79–81) a decrease in milk yield can be observed already 6 weeks before the clinically visible presentation of a lameness case. Hence in regard to milk yield, these cows are not standing out on average. This means that only a continuous assessment of the animals for lameness, for instance every fortnight, in conjunction with an evaluation of their performance immediately after calving would have produced the possibility to make a final assumption that high milk yield or high performance in the initial stage of lactation, respectively, entails a higher risk for lameness.

If cleanliness of the lower legs was compromised to the extent that distinct plaques of manure were present, the odds for lameness were increased. As lame cows spend a greater daily amount of time lying with shorter lying bouts (11, 82), this contamination of the lower legs may arise from increased exposure to excrements so that it would be rather a consequence of lameness. Also, an alternated lying behavior or an unphysiological lying position may further promote the contamination of the legs. As animals in tie stall facilities are constantly fixed in the same stall, they do not have the possibility to evade these conditions. On the other hand, contaminated legs may favor the development of lameness as the lower legs are exposed to increased bacterial contamination (9, 83–85). Urine and feces chemically impinge upon the integrity of the skin that may trigger the development of infectious claw pathologies. Interestingly, solid plaques of manure did not appear to be significantly associated with lameness in the final model. This might be the result of other protective factors attributable to deficient management that cover the influence of heavily contaminated legs. Hence, heavily contaminated legs (solid plaques of manure on the lower legs) may not have been necessary to increase the explanatory power of the model.

The presence of skin changes on the hock was associated with increased odds of lameness in accordance with previous studies and can be mainly traced back to three circumstances (37, 86, 87). Firstly, hock lesions can themselves be painful and hence cause lameness (88). However, this might apply to a minor percentage of cows as most cases of lameness can be traced back to pathologies of the claws (13, 22). Secondly, hock lesions may be a result of lameness. As lame cows are impaired in their ability to lie down and rise physiologically, they may collide with stall control elements which eventually gives rise to the development of lesions on the hock (87, 89). Furthermore, the quality of bedding and the amount of bedding material are other important factors in the context of lameness and hock lesions that may aggravate the situation. Also, as lame cows spent a greater amount of time lying (11, 82), their risk of developing hock lesions may increase due to abrasive properties of stall surface, low amount of bedding material or soiled bedding material (87, 89, 90). Finally, hock lesions and lameness are associated with similar factors that foster their occurrence (86, 91) which may be an important point when regarding their association.

Knowledge on the occurrence and importance of rib swellings has been scarce. They often rather represent an additional finding and may point to previous rib fractures. They typically occur between the 7 and the 9th rib at the transition from the boney part of the rib to the cartilaginous part (35, 92, 93). In the current study, they were highly associated with lameness. This association is plausible given the fact that lame animals have difficulties in rising and lying down as discussed previously. They hence may frequently slip or fall down harshly with the consequence of lesions of the ribs (35). Another hypothesis on the pathogenesis of rib swellings may be that lame animals tend to lean against dividers of their stalls and when they slip or try to lie down, their thorax collides with these elements (94). The association between lameness and rib swellings has previously been discovered but may need more research to discover the etiologic mechanisms. As rib fractures are likely to be very painful, their relevance to animal welfare is obvious.

The length of the stalls appeared as a factor associated with lameness in the final model. Both medium (> 158–171 cm) and short (≤ 158 cm) stalls increased the odds of lameness compared with long (> 171 cm) stalls. For a physiological lying and rising process, an adequately sized stall, which is the place of permanent inhabitation of a cow in tie stall housing, is of the utmost importance. Short stalls result in cows often lying down with parts of their body in the gutter area which frequently is either covered in manure or built as a grate. This is likely to have adverse effects of microbiological and physical integrity of the claws and facilitate the emergence of infectious and traumatic claw lesions. Short stalls also interfere with the cows' desire to lie down in a comfortable, well-bedded stall and hence significantly compromise the animals' well-being (95–97).

The currently available literature has presented equivocal opinions on the association between farm size and lameness. Whereas, evidence from a recent meta-analysis (20) as well as results from previous work (86, 98) suggest an association between increasing herd size and lameness as a result of less intensive surveillance of the individual animal, decreased availability of qualified staff or overstocking rather than a larger herd *per se*, other studies have yet observed lower lameness prevalences in larger herds (53, 77, 78). The latter studies suggest an increased level of professionalism, more personnel specifically trained for identifying lame cows and automated management elements. The current study suggests that a herd size of > 30 cows entails greater odds for the individual animal to be lame. We yet think this finding is ought to be interpreted cautiously as the general farm size was very low (range 4–61 cows). Nevertheless, this may be a perspective for future research to identify the role of herd size in dairy cow lameness especially in tie stall operations where lameness detection itself might be more challenging as outlined previously.

## Conclusions

The present study determined the prevalence of lameness in tie stall housed dairy cows and identified factors associated with lameness in this housing system. Housing conditions and elements of stall design are paramount in tie stall systems and in regard to lameness, they may possess an even more pivotal role in restrictive housing systems. Moreover, some aspects of housing and management are elements that allow for modification and improvement already in the short or the medium term. Following recommendations for stall design and management in these husbandry systems may be beneficial for both animal welfare and the prevalence of lameness. Furthermore, animal-level factors such as low body condition, higher parity, the presence of hock lesions and of rib swelling are important aspects in the context of dairy cow lameness which ought to be understood in order to tackle lameness problems and to improve animal welfare. Some of these factors may also require future investigations to better understand their inter-relationships especially in tie stall facilities.

## Data Availability Statement

The datasets presented in this article are not readily available because the study was initiated by a Federal Ministry. Even though data are anonymized, they are not allowed to be made available to subjects not involved in the initial study. Requests to access the datasets should be directed to Roswitha Merle, roswitha.merle@fu-berlin.de.

## Author Contributions

AO and RM initiated, conducted, supervised the study, and performed statistical analyses. AO drafted the manuscript with support from KJ, AT, and K-EM. AO, KJ, AT, and RM were involved in data cleaning, handling of the variables, and descriptive analyses. MF and K-EM contributed their professional expertise in the field and critically revised the manuscript. All authors have read and approved of the final manuscript.

## Conflict of Interest

The authors declare that the research was conducted in the absence of any commercial or financial relationships that could be construed as a potential conflict of interest.
